# Molecular and functional characterization of protease from psychrotrophic *Bacillus* sp. HM49 in North-western Himalaya

**DOI:** 10.1371/journal.pone.0283677

**Published:** 2023-03-30

**Authors:** Hina Mushtaq, Shabir Ahmad Ganai, Arshid Jehangir, Bashir Ahmad Ganai, Rubiya Dar

**Affiliations:** 1 Department of Environmental Science, University of Kashmir, Srinagar, Jammu and Kashmir, India; 2 Division of Basic Sciences and Humanities, Faculty of Agriculture, SKUAST-Kashmir, Sopore, Jammu and Kashmir, India; 3 Centre of Research for Development, University of Kashmir, Srinagar, Jammu and Kashmir, India; Annamalai University, INDIA

## Abstract

In this work, a psychrotrophic bacteria producing cold-active protease, was obtained from Dachigam National Park, an ecologically significant habitat in Western Himalayas owing to its varied endemic and endangered flora and fauna. This isolate was identified as *Bacillus sp*. HM49 via phenotypic, Gram staining, bio-chemical and 16S rRNA gene identification. Isolate HM49 when tested for proteolytic activity revealed prominent hydrolytic zone with the most production at 20 °C and pH, 8.0 post 72 h incubation. This enzyme was purified, enhancing its specific activity to 61.15 U/mg and its characterization studies revealed it to be a cold-alkaline protease being active in a wide pH (6.0–12) and temperature (5–40 °C) range. Amplification of CAASPR gene of HM49 was performed, followed by enzyme-substrate docking studies and MMGBSA providing details about its type, molecular weight validation as well as functional applications. The purified protease of HM49 was tested for laundry applications and the enzyme was found to be compatible with majority of the detergents tested. Its potential as an eco-friendly detergent additive was further validated by wash performance test as it effectively removed recalcitrant blood stains at a low temperature of 20 °C that could be beneficial for fine garments like silk which preferably need cold washing.

## Introduction

Microorganisms constitute a major portion of the biodiversity on planet, Earth playing some of the vital functions in various ecological processes which are essential for the sustenance of the life. Microorganisms are found in almost every natural habitat (air, soil, and water), with soil being the most preferred habitat mainly because it serves as a reservoir for diverse microorganisms [1]. Likewise, protease producing microorganisms are also found everywhere as they have been reported to be isolated from soil [2], wastewater and sludge [3], as well as undersea fumaroles [4]. In fact, some microorganisms can live in areas which are often hostile for the continuation of life having extreme physico-chemical conditions as in alkaline and saline lakes, hot and cold deserts, hot springs and ocean beds. Such extreme environments are thus most commonly colonized by extreme microorganisms, also known as extremophiles including cold-adapted, psychrotrophs. The microorganisms from these habitats are of great significance as they are a source of several commercial enzymes like, amylase, cellulase, lipase, and protease that can be utilized in different industrial applications such as food, detergent, leather processing, pharmaceutical industries, silk degumming, silver recovery and bioremediation purposes [5–7]. Among these enzymes, proteases are commercially most important and have widely been used for laundry applications as detergent additives mainly due to their excellent cost-ratio and efficient performance [8].

Proteases belong to hydrolase class of enzymes which are specific for the hydrolysis of the peptide bonds present in the polypeptide chain of amino acids to produce free amino acids and peptides [9]. Proteases, are thus those enzymes which are degradative in nature and shows great specificity and selectivity during protein modifications [10]. On the basis of their catalytic mechanism, proteases are grouped into exopeptidases which hydrolyse the proteins from their terminal and endopeptidases hydrolysing the proteins in the interior [11]. Proteases, on the basis of pH, are divided into acidic, neutral and alkaline [12]. Among them, alkaline proteases are the most important for industrial purposes mainly due to their capability to withstand higher pH conditions [13, 14].

Proteases form an integral component of life on earth, and are ubiquitously found in plants, animals, and several species of microbes such as bacteria, fungi, and yeast [9]. On account of their high yield, genetic manipulation, ease in culturing and downstream production, as well as immense industrial applications, bacteria, mainly the *Bacillus* group, are the most active and dynamic extracellular alkaline protease producers in the industrial sector and thus constitute the major source of commercial proteases [15]. It is in this context that alkaline proteases from several *Bacillus* sp. have been extensively studied, however, such proteases have been found to have restricted activity at low temperatures [16]. Therefore, attempts are being continuously made to identify novel cold-active protease producing bacteria which could meet the increasing industrial demand mainly for various cleaning and laundry operations.

Cold-active enzymes and proteins from psychrotrophic microorganisms have various biotechnological applications [17] with their proteases emerging as an effective alternative in detergent industry on account of their high activity at lower temperatures that would not only minimise the consumption of energy but also the wear and tear inflicted on the textile fibres. The utilization of such cold-active proteases in detergent formulations would thus be advantageous for cold washing thereby saving energy and providing financial benefits [18, 19].

Various cold-adapted proteases have been previously obtained from several colder environments including soils in Turkey [20], surface sediments in Greenland [21], deep-water mud of East Indian Ocean [22] as well as from different soil samples in Indian Himalayan region like Lahaul and Spiti [23] and Gangotri glacier [24]. However, till date no study has been carried out on the cold-tolerant protease producing bacteria from the North-Western Himalayan range, mainly the region encompassing through Kashmir, which is a composite of varying climatic zones with diversified high-altitude peaks and contrasting soil textures containing rich diversity of endangered plants, animals as well as microorganisms [25, 26]. Therefore, this study’s objective comprised of the (i) isolation and identification of an efficient protease secreting psychrotrophic bacteria from the soils of Dachigam National Park (DNP) (ii) characterization and functional application of this psychrotrophic bacterial enzyme for its utilization in developing green detergent additive.

## Material and methods

### Sample collection

Soils were sampled on a seasonal basis (i.e., Spring, Summer, Autumn, and Winter) for two years from a grassland site in lower part of Dachigam National Park (henceforth DNP) having a geographical latitudinal extent of 34°09.074^2^ N and a longitude of 74°55.392^2^ E at an altitude of 1743 m. Soil samples were collected in sterile vials, kept in an ice-box and transported to laboratory for further analysis [27].

### Isolation of cultivable soil bacteria

For the isolation of bacteria, the samples were mixed thoroughly; sieved and about 1g of soil was used for standard serial dilution technique [28]. Aliquots of 100 μl were then inoculated on prepared plates of nutrient agar that was pre-autoclaved for 15 min at 121 °C [29]. The inoculated plates were placed at 37±2 °C in inverted positions for 24–48 h. Single isolated colonies were used for obtaining pure cultures of the bacteria by sub-culturing repeatedly through streak plate method. Purified bacterial stains were stored at 4 °C in nutrient agar slants for future use.

### Preliminary screening for protease producing bacteria

All the isolated pure bacterial strains were investigated for their protease enzyme activity on skimmed-milk medium that was prepared using agar 15 gl^-1^, dextrose 1 gl^-1^, skim milk powder 28 gl^-1^, tryptone 5 gl^-1^ and, yeast-extract 2.5 gl^-1^, having pH 7.0 ± 0.2. The media was autoclaved for 10 min at 121 °C, cooled, dispensed into plates and solidified. After inoculating a loop-full of inoculum for every bacterial isolate on the prepared plates, they were placed in incubation at 37±2 °C for 24 h [30]. On completion of the incubation period, the zone of hydrolytic clearance surrounding the colonies of each isolate depicting protease production were checked and the colony showing highest hydrolytic zone was selected for further study.

### Primary screening of proteolytic bacteria, HM49 on different media at different incubation temperatures

The primary screening for proteolytic activity by isolate, HM49 was carried on three different media namely casein agar; gelatin agar and skimmed milk agar, each having two sets of pH i.e., 7.0 and 8.0. The media was prepared using agar (15 gl^-1^), yeast extract (1 gl^-1^), peptone (4 gl^-1^) containing 12 gl^-1^ (w/v) of each casein, gelatin and skim milk as a protein source. After sterilisation and solidification of media, the petri-plates (in triplicates) were inoculated and kept in an inverted position under varied temperatures of incubation (10 °C, 20 °C, 30 °C, and 40 °C) for 24–72 h whereby region of hydrolytic clearance around the bacterial colony was examined every 24 h.

### Secondary screening of isolate, HM49 for protease production

The basal medium was prepared at optimized pH 8.0 using 5 gl^-1^ skim milk; along with 10 gl^-1^ glucose; 10 gl^-1^ sodium carbonate (Na_2_CO_3_); 7.5 gl^-1^ peptone; 5 gl^-1^ sodium chloride (NaCl); 2gl^-1^ potassium dihydrogen phosphate (KH_2_PO_4_); 2 gl^-1^ magnesium sulfate (MgSO_4_); and 0.1 gl^-1^ ferrous sulphate (FeSO_4_.7H_2_O) in distilled water, sterilised by autoclaving and cooled. The extracellular protease enzyme production by isolate HM49 was assessed by aseptically adding 5% (v/v) of overnight bacterial broth culture having OD_550_ of about ≈0.2 to 50 ml of prepared media in sterilised 250 ml Erlenmeyer’s flask. The inoculated flasks were then kept in a rotary shaker (120 rpm) for 72 h at 20 °C (i.e., the optimized fermentation time and temperature respectively).

### Extraction of the crude proteases

After the incubation period, the culture in each flask containing protease procured from the cultivation of bacterial isolate, HM49 was centrifuged (4 °C) at 10000 rpm (8400 x g) for about 15 min. The pellet was discarded and the cell-free culture supernatant consisting of crude enzyme was kept in a refrigerator at 4 °C and utilised for further analysis. Protein content and protease activity assay of the culture supernatant were evaluated.

### Purification of protease from isolate, HM49

Solid ammonium sulphate precipitation method (70%) was utilized for precipitating the protease [31]. After dissolving the standard amount of ammonium sulphate in the culture supernatant, it was centrifuged (10,000 rpm) for 15 min at 4 °C. Pellet retrieved after precipitation was dissolved in 30 mM tris-buffer having a pH of 8.0 and dialyzed against the previously used buffer. This fraction is partially purified enzyme (PPE) which was then used for characterization and application study. The protein content and protease activity of precipitated as well as dialyzed enzyme were estimated.

### Protein assay

The protein concentration in enzyme (mg ml^-1^) was evaluated taking bovine serum albumin (BSA) as a reference following protein estimation method [32] and the absorbance was read at 700 nm.

### Protease activity assay

The supernatant devoid of bacterial cells containing crude enzyme was utilized for the protease activity assay (U/ml) with casein as substrate. The proteolytic activity was examined as per the standard protocol [33] with a few modifications whereby 0.65% casein (w/v) in 0.1M buffer of tris-HCl together with enzyme was put at 20 °C for half an hour. A blank was also run simultaneously. The reaction was stopped by the addition of 10% (w/v) trichloroacetic acid (TCA) followed by half an hour incubation at 37 °C. The sample as well as blank were then centrifuged (4 °C) at 10000 rpm for 10 min. This was followed by adding 0.4 M sodium carbonate and 1N Folin-Ciocalteau reagent to the supernatant previously taken in a fresh test tube. This mixture was gently stirred, incubated for thirty minutes at 37 °C and its A_660_ was noted. The assay temperature as well as pH were amended whereby protease was kept for incubation in the pH range of 6–12 and the temperature ranging 10–40 °C respectively. Thus, for the assay of this protease, the activity was computed at a set pH of 8.0 and a temperature of 20 °C.

For calculations, tyrosine standard curve was utilised to calculate the released amount of tyrosine. An activity unit of enzyme was elucidated as the enzyme quantity needed for hydrolysing casein to liberate 1.0 μmol of tyrosine per ml per minute in typical conditions of assay. However, its specific activity was determined by dividing enzyme units (U) over its protein concentration (mg per ml). Therefore, activity was designated in units (U) whereas the enzyme specific activity was marked as activity per milligram of protein (U/mg).

### Optimization of factors for protease production employing one variable at one-time approach

#### Optimization of pH

For the evaluation of the most appropriate pH for protease generation by the isolate HM49, the basal media (100 ml) was prepared as mentioned above in five Erlenmeyer flasks (250 ml), wherein the pH was adjusted in a range from 6.0 to 10.0. After inoculating these flasks with 5% (v/v) of overnight bacterial broth having an A_550_ of ≈0.2, they were incubated (72 h) in a rotary shaker at 20 °C followed by determination of their activities.

#### Optimization of temperature

Autoclaved basal medium was put in five sterilised Erlenmeyer flasks (100 ml in each), inoculated with fresh 5% (v/v) of bacterial culture of 24h with an A_550_ of about ≈0.2 and kept at 10, 15, 20, 25 and 30 °C (72 h) at 120 rpm in an orbital shaker so as to check the temperature influence on production of enzyme by this isolate. After the incubation period, their enzyme activities were evaluated.

#### Optimization of time

To 100 ml sterilised medium kept in an Erlenmeyer conical flask (250 ml), about 5% (v/v) of fresh bacterial inoculum (A_550_ ≈0.2) was added. The fermentation of enzyme was conducted from 24 h up to 120 h at its optimal temperature (20 °C), and activity of the produced extracellular protease was estimated at 12 h interval on completion of 24 h incubation.

### Enzyme characterization

#### Effects of pH on protease activity of isolate, HM49

Influence of pH on the activity of enzyme was checked with buffers (0.1M) ranging 6–12 and included phosphate buffer for pH 6.0–7.0, tris-HCl buffer for pH 8.0–9.0 and glycine-NaOH buffer for pH 10.0–12.0. Using 0.65% casein (proteinaceous substrate), activity was evaluated by placing the reaction mixtures (20 °C) for an hour in the respective buffer. The activity was computed as measured activity in relation to activity obtained at optimum pH i.e., 100% and was expressed as relative activity (%).

#### Effects of temperature on protease activity of isolate, HM49

This was assessed using 0.65% casein in 0.1M buffer of tris-HCl (pH 8) at temperatures from 05–40 °C. Relative enzyme activity (%) was computed considering activity at its ideal temperature (100%).

### Identification of isolate HM49

#### Phenotypic identification

The macro-morphological colony characteristics of the isolate, HM49 was observed using Bergey′s manual [34] followed by Gram staining and examination for cellular morphology and arrangement under fluorescent microscope (Olympus-1X71, USA).

#### Biochemical identification

The utilization of carbohydrate was checked using test kits (HiCarbohydrate Kit-KB009by HiMedia, India) following manufacturer’s instructions. A pure single colony of isolate, HM49 was put in about five ml of freshly prepared Brain Heart broth (HiMedia), kept at 35–37 °C till the inoculum turbidity reached OD_620_ ≥ 0.5. Each well in the kit was then inoculated with 50 μl of this broth by virtue of surface-inoculation and kept for 18–24 h at 35 °C ± 2 °C. In addition, isolate HM49 was examined for the secretion of enzymes, namely, amylase, cellulase, and lipase using starch agar, carboxymethyl cellulose (CMC) agar, and tributyrin agar media respectively.

#### Molecular identification

16S ribosomal RNA analysis was carried out by extracting the DNA of bacterial isolate HM49 using QIAprep^®^Spin Miniprep Kit (Catalog ID. 27104, by QIAGEN laboratories) following the manufacturers protocol with slight modifications. The extracted genomic DNA was utilized as a template for 16S rRNA gene amplification study. Amplification was performed by PCR (polymerase chain reaction) in a thermo-cycler (CG Palm Cycler by Genetix Biotech Asia Pvt. Ltd) using bacterial primer set 27F (5'- AGA GTT TGA TCC TGG CTC AG-3') and 1492R (5'-GGT TAC CTT GTT ACG ACT T-3') that were synthesized by IDT (Integrated DNA Technologies) and yielded a PCR product of about 1.5 kb. The amplification process was accomplished in a volume of 50 μl reaction mix and the cycling parameters comprised of 5 minutes at 94 °C (initial de-naturation) followed by 30 cycles, each of 1 minute at 94 °C (de-naturation), 45 seconds at 55 °C (annealing), 2 minutes at 72 °C (extension) and 10 minutes at 72 °C (final extension). For negative control, ultrapure water (MilliQ) was used in place of exogenous template. An amplified product of the expected size, approximately 1500 nucleotides (1.5 kb) was observed which was examined and confirmed via gel electrophoresis on 1.5% agarose gel with ethidium bromide stain in 1X-TAE (tris-acetate-EDTA) buffer and the banding pattern was visualized using UV illumination in a GEL DOC/Bio-imaging System. A 100 base-pair DNA ladder (ThermoScientific Genruler) was employed as a standard DNA marker. The PCR product was dispatched to AgriGenome Lab, Kerala to purify the amplicon and its subsequent Sanger DNA sequencing.

#### BLAST analysis of 16S rRNA gene sequence of isolate, HM49

Basic local alignment search tool for nucleotides (BLASTn) was employed to identify the retrieved nucleotide sequence by determining the phylogenetic neighbours from the nucleotide databases of National Centre for Biotechnology Information (NCBI).

#### Phylogenetic tree construction of isolate, HM49

Representative nucleotide sequences of similar neighbours in BLAST analysis were obtained and aligned through Clustal-W of MEGA 7. The phylogenetic tree of evolution was prepared by neighbour-joining employing correction factor of Maximum Composite Likelihood with a bootstrap value of one thousand replicates.

### Antibiotic susceptibility test

Isolate, HM49 was tested for susceptibility of various antibiotics (S1 Table) by disc diffusion method using Hexa G-plus discs (HX001 and HX003 by HiMedia, India) having different antibiotic concentration (Clinical and Laboratory Standards Institute, CLSI). Single colony of this bacteria was inoculated in 5 ml Nutrient broth followed by incubation at 35 ± 2 °C till the inoculum turbidity (OD_600_) reached ≈0.2. About 100 μl (0.1 ml) of this bacterial broth culture was spread on the prepared Mueller Hinton agar (HiMedia, India) media plates and the antibiotic discs were aseptically dispensed using sterilised forceps. The plates were kept in incubation (37 ± 2 °C) for 16–18 h after which they were checked for the antibiotic sensitivity. The formation of clear areas around a disc depicted sensitivity of the inoculated bacteria towards that particular antibiotic. However, any growth around the discs indicated the resistance of the bacteria to those peculiar antibiotics.

### Amplification of serine protease gene

For amplifying the gene encoding the cold-active alkaline serine protease from isolate HM49 (CAASPR-HM49), a primer set (CAASPR forward: 5'-CGC GGA TCC GTG GGT TTA GGT AAG AAA TTG-3' and CAASPR reverse: 5'-GCG TCG ACT TAC AAT CCG ACT GCA TTC C-3') was designed by Primer Blast Tool (NCBI) using the genetic information of *Bacillus velezensis* available on GenBank, NCBI. Previously extracted DNA was taken as PCR template wherein cycling conditions comprised of initial denaturation of five minutes at 94 °C with 35 cycles of each having, denaturation at 94 °C for one minute, annealing at 50.5 °C for forty-five seconds, extension at 72 °C for two minutes and final extension at 72 °C for ten minutes.

#### Protein sequence analysis and properties of CAASPR-HM49

The translated protein sequence was blasted (BLASTp, NCBI) and the conserved domain of CAASPR-HM49 was predicted by Conserved Domain Database (CDD) tool of NCBI (https://www.ncbi.nlm.nih.gov/cdd). Evolutionary phylogenetic relationships tree was constructed by MEGA 7 following neighbour joining method. On the other hand, the multiple sequence alignment was made using COBALT, NCBI (https://www.ncbi.nlm.nih.gov/tools/cobalt/re_cobalt.cgi). The web-tool, Expasy’s ProtParam was utilized to assess the features of CAASPR-HM49 such as molecular weight, and isoelectric point (pI) (https://web.expasy.org/protparam/).

#### Model generation and refinement

The model of CAASPR-HM49 was generated by taking the assistance of GalaxyTBM. For generating the model from a given protein sequence, this software involves two stages. In the first stage, HHsearch results are rescored, core sequences of target and templates are aligned by PROMALS3D and then model building is performed by MODELLERCSA. Following this, the second stage begins in which the loop or terminus areas are spotted. These regions are remodelled by way of an optimization reliant refinement protocol [35, 36]. In the present study PDB ID: 3AFG served the purpose of template. Among the various models generated for a single protein sequence, the most appropriate model was picked and subjected to further refinement. This refinement was done by taking the advantage of another tool known as GalaxyRefine. Through this tool regional as well as global qualities of model were strongly tuned [37].

#### Validation of refined model

As usual, the refined model of CAASPR-HM49 was validated through different tools. PROCHECK was used for testing the stereochemical quality of the above-mentioned model. Verify 3D was employed for assuring 3D-1D compatibility while the comparison of model to experimentally determined structures was done by using PROSA [38–41].

#### Preparing receptor and ligand coordinates

From PubChem, the coordinates of lactose (PubChem CID: 3037558) were acquired [42]. This structure was made suitable for molecular docking using a LigPrep tool. Default parameters were maintained while the preparation of lactose. The refined model of subtilisin like protease was made fertile for docking through Protein Preparation Wizard [43]. This component of Schrödinger suite fills missing atoms, side chains and loops. Apart from this, useless items accompanying the protein of interest were also expunged. Water molecules not fulfilling the retention criteria were removed. Protein Preparation wizard not only optimizes the structure but also minimizes it effectively. After minimization, the active site was confirmed using ScanProsite tool. These residues were employed for grid-specification through Glide [44, 45].

#### High-exactitude molecular docking and binding free energy evaluation

The prepared lactose molecule was docked against the active site of CAASPR-HM49 using the high-exactitude molecular docking protocol [46]. The docking was done through Glide tool and the best pose was picked based on more negative value of docking score. For assessment of binding free energy, the docked complex was subjected to molecular mechanics generalized born surface area estimations. This method measures multiple energies and from those calculations the binding free energy was estimated using the equation described in earlier studies [47]. Default solvation model was maintained throughout energy estimations.

#### Atomic scale interaction between the lactose and CAASPR-HM49

This was generated by taking the advantage of newly emerged protein ligand interaction profiler. This profiler helps in gaining thorough information about various non-covalent interactions occurring between the ligand and receptor. Among these interactions, hydrogen bonds, salt bridges, pi-pi, pi-cation and hydrophobic interactions are prominent [48]. CAASPR-HM49 in docked condition with the best pose of lactose in a feasible format was used as an input for generating the aforesaid profile.

### Application of CAASPR-HM49

#### Commercial detergent compatibility test

Some commonly used commercial detergents such as Ariel (P&G Ltd.), Fena (G.C.I.S. Ltd.), Ghadi (R.S. Pvt. Ltd.), Henko (H.S.I. Ltd.) and Rin (H.U. Ltd.) were used (7 mg per ml final concentration) in order to perform the compatibility test for enzyme from HM49. Prior to their use for the test, the endo-genous proteases of these detergents were deactivated by incubation at 80–100 °C for 1h. The detergent compatibility test was performed by incubating the PPE with each of the detergent solution (1:4 ratio) at 20 °C for 1h after which their remaining activity was determined. Activity of control (PPE alone) was considered to be 100%.

#### Cold-wash performance

The application of this protease enzyme for laundry industry with its two most compatible detergents was assessed by cold water wash performance for removing the tough blood stains using pieces of white cotton cloth of 4 x 4 cm^2^ dimension. The cloth pieces were stained (SC) using blood and dried in an oven for 5 minutes at 95–100 °C. They were then kept in separate plates and a set of below treatments (in triplicates) was devised:

- 20 millilitres of Ghadi detergent solution + SC
- 20 millilitres of Ariel detergent solution + SC
- 20 millilitres of Ghadi detergent solution + 0.5 ml enzyme+ SC
- 20 millilitres of Ariel detergent solution + 0.5 ml enzyme + SC
- 20 millilitres of tap water (T.W.) + 0.5 ml enzyme + SC
- 20 millilitres of tap water (T.W.) + SC as control

These plates were placed at 20 °C for twenty minutes and regularly taken out for visual examination of the stains. On completion of the time, the SC pieces were properly cleansed with tap water, air-dried and carefully checked for stain removal. The untreated blood cloth was considered as the control.

#### Management of wastes: Chicken feather disintegration test

Whole chicken feather obtained from a local poultry was washed thoroughly with tap water to remove contamination (if any) followed by distilled water rinse and autoclaving. The feather was air-dried and the disintegration test was carried out at an optimized test temperature of 20 °C following Mushtaq et. al [37].

### Statistical analysis

The data was initially analysed using Microsoft Excel 2019 (Microsoft Corporation), and results designate the mean standard deviation (Mean ± SD) of three independent replicates after which the Tukey’s test was performed. The results were taken as statistically significant for P values ≤ 0.05 as depicted by different letters above the bars.

## Results

### Isolation and preliminary check of isolated bacteria for proteolytic activity

Fifteen (15) morphologically varying bacterial isolates were isolated from the grassland site of lower DNP, out of which 08 bacterial isolates were producing protease as reflected by the clear hydrolytic areas around their colonies (Table 1). Out of these protease positive bacteria, isolate HM49, showed maximum hydrolysis around its colony on the skim milk agar plate due to which it was selected for further analysis.

**Table 1:** Preliminary screening of isolated bacteria for their proteolytic activity.

Isolate ID	Mean Hydrolytic Zone of Clearance, H (in mm)	Mean Colony Diameter, C (in mm)	Mean H/C ratio (in mm)
HM49	15.00 ± 2	4.33 ± 0.58	3.50 ± 0.66
HM50	6.00 ± 1.73	4.00 ± 1.73	1.78 ± 0.96
HM52	6.33 ± 1.53	5.67 ± 1.15	1.11 ± 0.10
HM53	7.00 ± 2.00	5.00 ± 1.73	1.57 ± 0.78
HM56	14.00 ± 1.00	4.33 ± 0.58	3.25 ± 0.25
HM57	15.00 ± 1.00	10 ± 2.00	1.55 ± 0.36
HM61	8.00 ± 1.00	6.67 ± 1.15	1.21 ± 0.11
HM88	8.00 ± 1.00	4.00 ± 1.73	2.28 ± 0.98

### Primary screening of proteolytic bacteria, HM49 on different media at different temperatures

**Fig 1:**
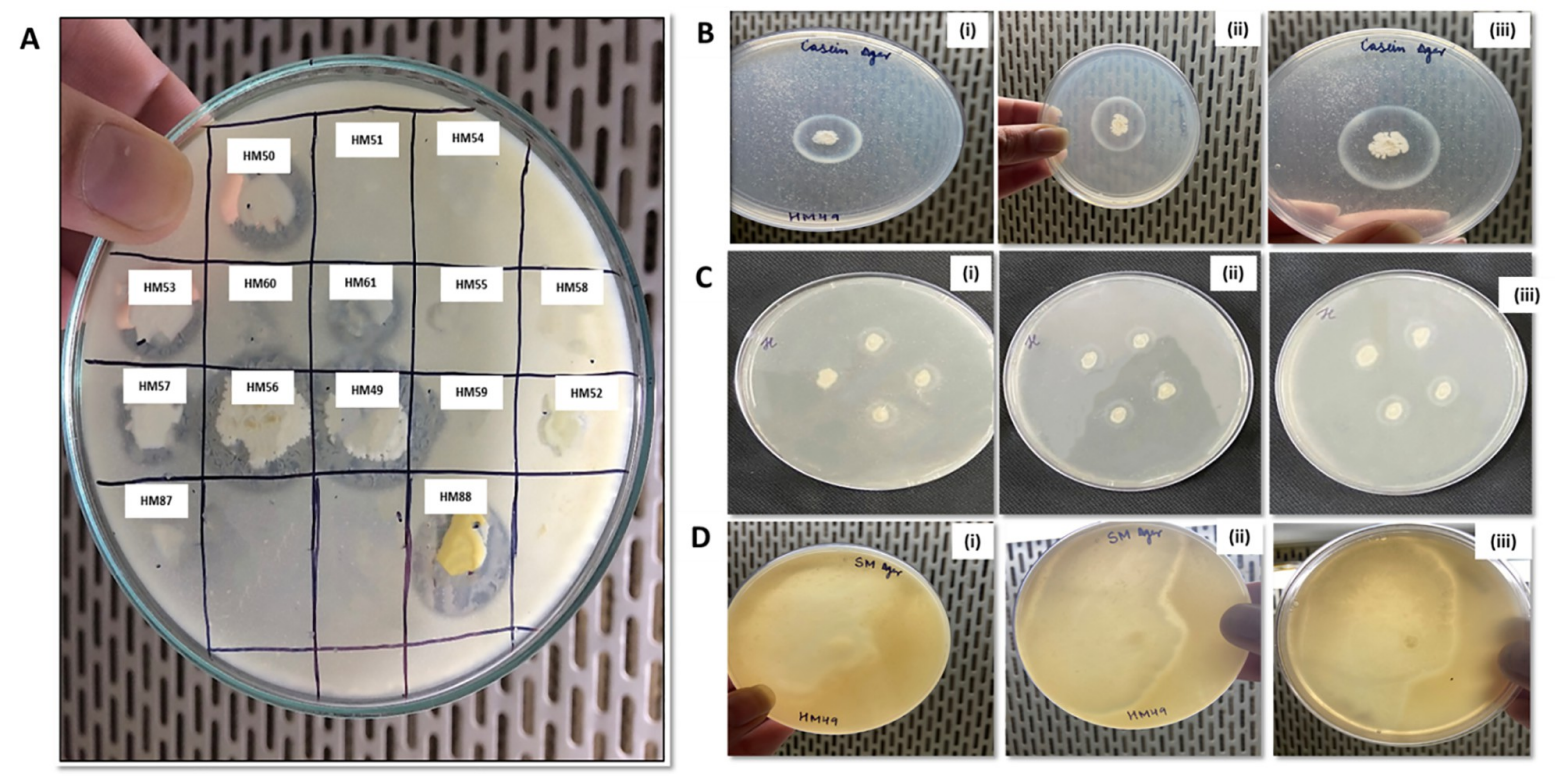
Screening of bacteria for its proteolytic activity- preliminary screening of isolated bacteria for their proteolytic activity on skim-milk agar (A); primary screening of *Bacillus* sp. HM49 on casein (B), gelatin (C) and skimmed milk (D) agar media at a temperature of 20 °C (pH 8.0) after (i) 24 h, (ii) 48 h, and (iii) 72 h incubation time.

Primary screening of isolate HM49 on casein, gelatin and skimmed milk agar media plates at pH (7 and 8) suggested notable hydrolysis all over its colonies after 24 h incubation at a temperature of 20 °C (Fig 1) on media plates (pH 8.0) containing skimmed milk as a source of protein (Table 2).

**Table 2:** Primary screening of proteolytic bacteria, HM49 on different media at different temperatures.

Protein Source	Incubation Temperature (in °C)	Incubation Time (in h)	Mean Zone of Clearance, *H/C ratio (in mm)
Casein	10	24	2.52 ± 0.32
		48	1.55 ± 0.13
		72	1.67 ± 0.15
	20	24	6.08 ± 0.14
		48	3.77 ± 0.36
		72	3.52 ± 0.11
	30	24	4.27 ± 0.12
		48	2.47 ± 0.12
		72	2.14 ± 0.19
	40	24	2.69 ± 0.13
		48	1.84 ± 0.07
		72	1.80 ± 0.05
Gelatin	10	24	1.45 ± 0.28
		48	1.31 ± 0.07
		72	1.42 ± 0.04
	20	24	3.38 ± 0.40
		48	2.22 ± 0.20
		72	2.48 ± 0.10
	30	24	2.82 ± 0.17
		48	2.12 ± 0.13
		72	2.06±0.19
	40	24	2.07 ± 0.12
		48	1.52 ± 0.11
		72	1.61 ± 0.10
Skimmed Milk	10	24	7.22 ± 0.51
		48	5.66 ± 0.44
		72	4.21 ± 0.47
	20	24	14.78 ± 0.39
		48	8.48 ± 0.22
		72	7.09 ± 0.41
	30	24	10.08 ± 0.14
		48	7.11 ± 0.10
		72	5.15 ± 0.22
	40	24	9.00 ± 0.25
		48	6.67 ± 0.17
		72	4.49 ± 0.25

*H = Hydrolytic Zone of Clearance (in mm)

C = Colony Diameter (in mm)

### Protein estimation and protease activity

The cell free culture supernatant of isolate, HM49 obtained after centrifugation was used as the crude protease enzyme source, which was then analyzed for its protein content and protease activity using standard spectrophotometric methods. Precipitation with solid ammonium sulphate and dialysis was utilized for the purification of crude protease of isolate, HM49. After every purification step while the total activity decreased, the specific activity on the other hand increased to 61.15 U/mg with a yield of 51.6% and 3.19 fold purification (Table 3).

**Table 3:** Protein purification of protease enzyme from isolate HM49.

Purification Step	Total Protein (mg)	Total Activity (U)	Specific activity (U/mg)	Yield (%)	Purification fold
Culture Supernatant	31.91	610.95	19.14	100	1
Precipitated Enzyme	11.72	456.97	39.00	74.8	2.04
Dialyzed Enzyme	5.16	315.20	61.15	51.6	3.19

### Optimization of enzyme producing conditions (pH, temperature, incubation time)

**Fig 2:**
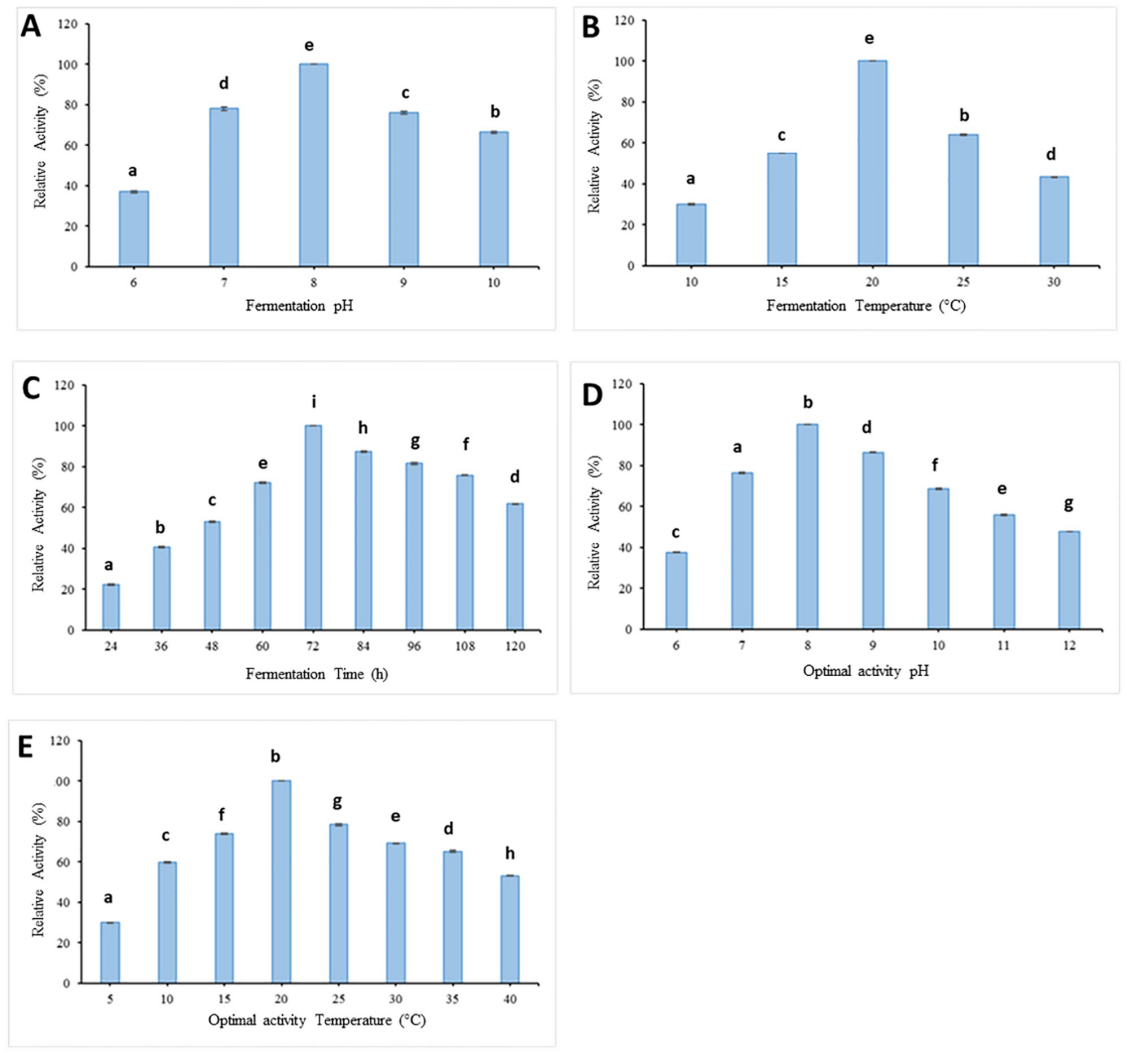
(A, B, C). Optimization of protease production by isolate HM49 (2A- fermentation pH; 2B- fermentation Temperature; 2C-fermentation Time). Isolate HM49 depicted optimal enzyme production in pH 8.0 at 20 °C post 72h incubation period. The data in figures represent mean standard deviation (Mean ± SD) of three independent replicates with different letters above the bars indicating significant enzyme activity at p = <0.05 significance level. (D, E). Effects of varying pH and temperature on protease activity. Isolate HM49 recorded its protease enzyme activity from pH 6 (lowest) reaching its optimum at pH 8 followed by a gradual decrease up to pH 12 (2D). Temperature studies revealed its dynamism broadly in 5°C (lowest) to 40 °C range with its optimum activity being recorded at 20 °C (2E). The data in figures represent mean standard deviation (Mean ± SD) of three independent replicates with different letters above the bars indicating significant enzyme activity at p = <0.05 significance level.

The optimization study revealed that the isolate HM49 produced the protease enzyme over 6.0–10 pH (Fig 2A) showing significant enzyme activity at pH 8 (p<0.05), thereby clearly reflecting that the alkaline medium was favourable for ample enzyme production. This isolate also produced the protease enzyme successfully in a varied range of temperatures (10–30 °C) reaching a significant production at 20 °C (p<0.05) right after 24h incubation (Fig 2B) which increased continuously up to 72h optimum (p<0.05) with a gradual decline upon further prolongation in time (Fig 2C).

### Enzyme characterization

#### Influence of pH on enzyme activity of isolate, HM49

pH effects over the proteolytic activity of isolate HM49 was observed at 0.65% substrate concentration using various pH buffers whereby the result suggested that it remained active over the pH range of 6–12. The least protease activity, 37.6% was recorded at pH 6, while pH 7, 9, 10, 11, 12 had 76.44%, 86.49%, 68.79%, 55.89% and 47.78% respectively relative to the highest significant activity (p<0.05) of 100% observed at pH 8 (Fig 2D).

#### Effects of temperature on protease activity of isolate, HM49

Study on the temperature profile of protease enzyme from isolate HM49 measured at 0.65% substrate concentration and pH 8 revealed its activeness over varied temperatures ranging 5–40 °C. The lowest relative enzyme activity of 29.99% was observed at 5 °C which increased continuously with a significant (p<0.05) optimum (100%) at 20 °C after which it decreased reaching 53.18% at 40 °C (Fig 2E).

### Identification of isolate, HM49

#### Phenotypic and biochemical identification

The Gram-positive proteolytic isolate, HM49 obtained from the grassland soils of DNP had a circular, cream coloured colony as depicted on nutrient agar plates (S1A and S1B Figs). The detailed colony characteristics of isolate HM49 along with its Gram staining properties are given in S2 Table.

Biochemical characterization depending on the carbohydrate utilization (S1C Fig) revealed that isolate HM49 utilized most of the monosaccharides namely dextrose, fructose, galactose, L-arabinose, mannose, sorbose, and xylose, however, it was not able to use any monosaccharide derivative except esculin from the test-kit. This isolate successfully utilized several di-, tri- and poly-saccharides such as lactose, melibiose, sucrose, maltose, raffinose, trehalose and inulin. In addition, isolate HM49 was capable of utilizing sugar alcohols like arabitol, mannitol and sorbitol, besides which citrate, sodium gluconate, ɑ-Methyl-D-glucoside and ɑ-Methyl-D-mannoside were also utilized by it (S3 Table). Although the isolate, HM49 was capable of producing amylase and lipase enzymes as indicated by prominent hydrolytic areas surrounding its colonies however, it was unable to produce cellulase enzyme (S1D–S1F Figs).

#### Molecular identification

**Fig 3:**
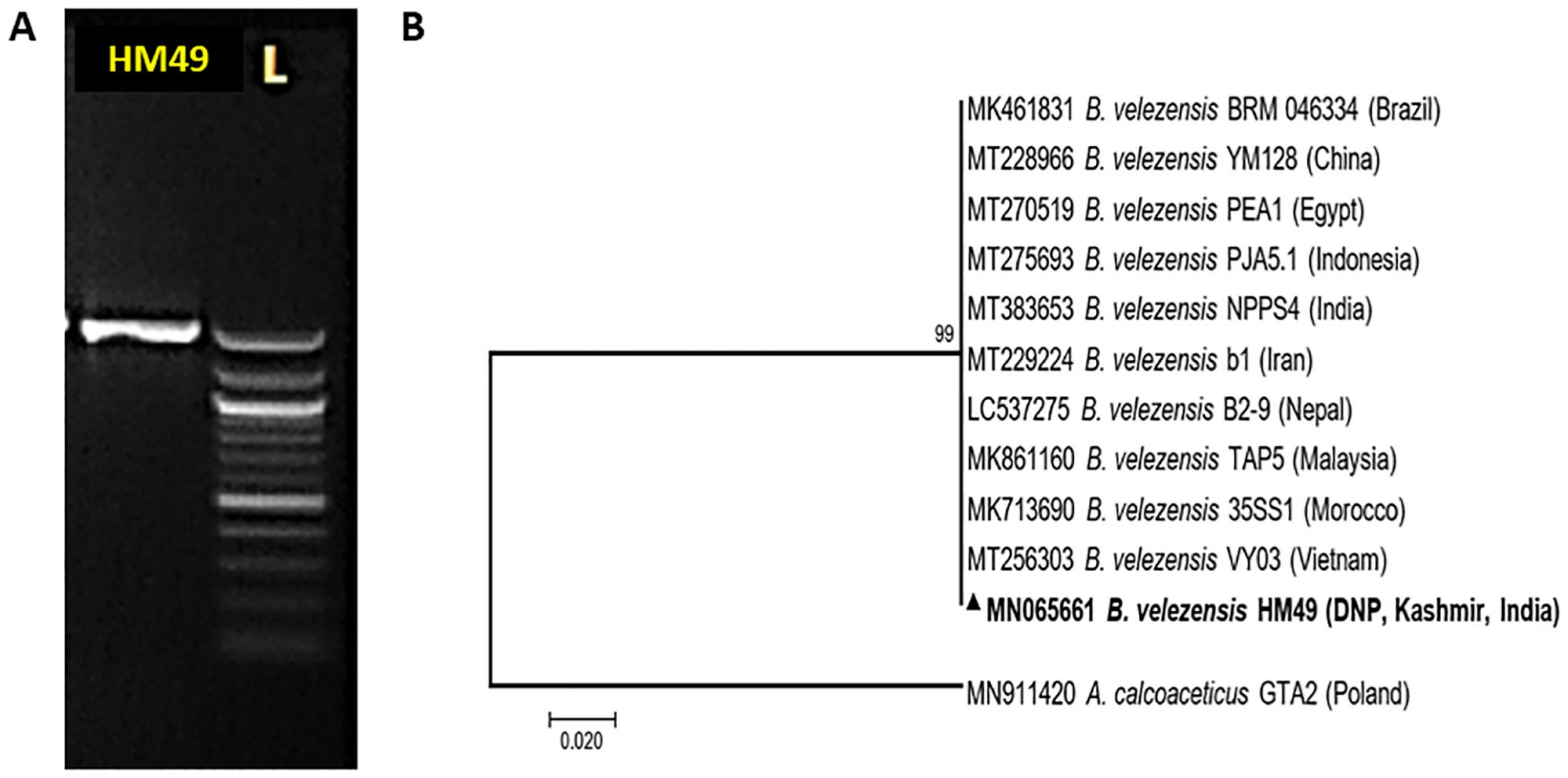
16S rRNA gene-based molecular identification of *Bacillus velezensis* HM49. Representative electrophoretic image of amplified PCR product (A); evolutionary neighbor-joining phylogenetic tree (B).

16S rRNA gene analysis, mainly used for the identification of prokaryotes was carried out retrieving a nucleotide sequence which when searched on NCBI databases using BLASTn showed similarity to sequence identity of *Bacillus velezensis*. It was then deposited to GenBank (http://www.ncbi.nlm.nih.gov/nuccore/MN065661). Evolutionary proximity of isolate HM49 with species of *B*. *velezensis* was obtained by phylogenetic tree construction using MEGA 7 (Fig 3 and S2 Fig).

### Antibiotic susceptibility test

The antibiotic susceptibility test results revealed that the isolate HM49 was sensitive to all the antibiotics under consideration i.e., aminoglycoside (gentamicin), cephalosporin’s (cefoxitin, ceftazidime, cephalothin), fluoroquinolone (ofloxacin), macrolide’s (clindamycin and erythromycin), penicillin’s (amoxiclav, oxacillin and penicillin-G), teicoplanin, and vancomycin (S3 Fig).

### Sequence analysis and properties of serine protease gene

**Fig 4:**
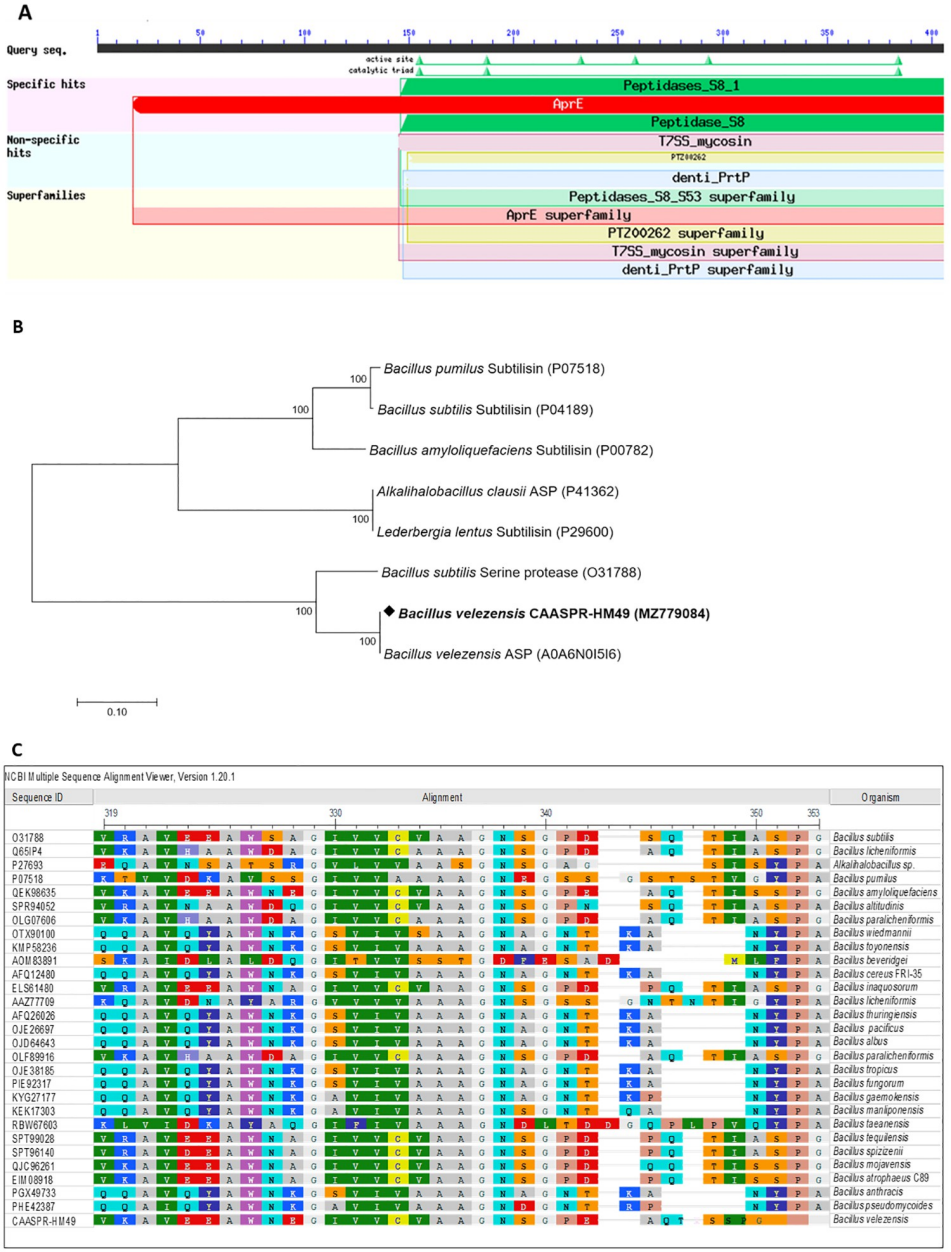
(A, B). Conserved domain prediction of CAASPR-HM49 protein (A). Evolutionary phylogenetic relationships of CAASPR-HM49 with other peptidases employing the neighbor-joining method (B). (C). Multiple sequence alignment of CAASPR-HM49 with several other typical proteases from the S8 family of extracellular subtilisins.

The extracellular cold-active alkaline serine protease (CAASPR) gene was amplified (~1326 bp) from the genomic DNA of *B*. *velezensis* HM49 (S4 Fig) and submitted in GenBank, NCBI under MZ779084 accession number. The conserved domain of CAASPR-HM49 was predicted (Fig 4A) after which the protein sequence of CAASPR-HM49 was checked with available proteases on MEROPS, NCBI and UniProt databases. The results of which revealed its close proximity with the serine peptidase family (S8A) as reflected in the phylogenetic tree (Fig 4B) and multiple sequence alignment (Fig 4C) of CAASPR-HM49. While its molecular weight was predicted to be ≈48 kDa, the computed theoretical isoelectric point (pI) on the other hand, was about 5.98.

#### Model quality was certified by multiple validation methods

**Fig 5:**
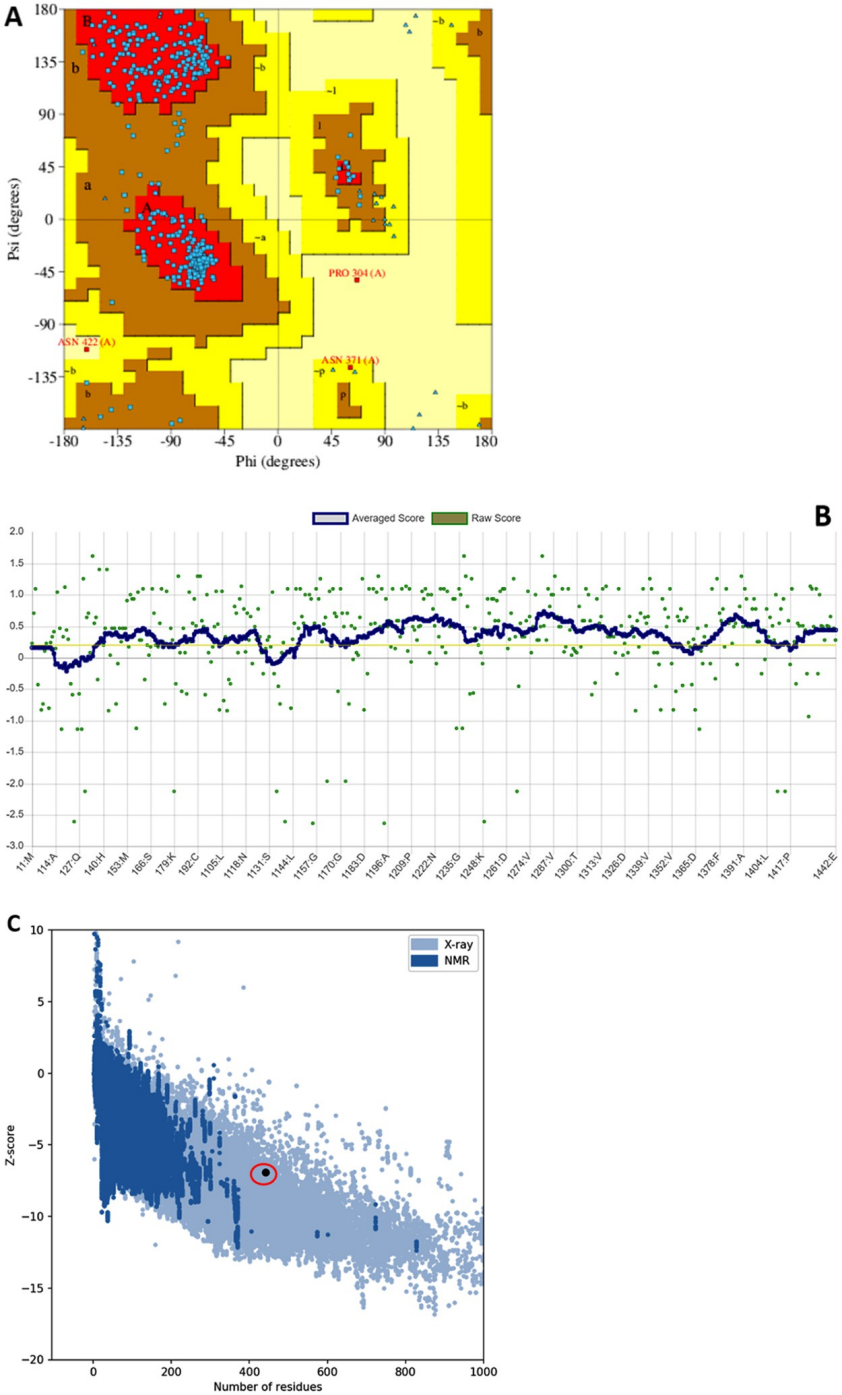
(A). Ramachandran plot of CAASPR-HM49 subtilisin like protease refined model. Above 90% residues were spotted in most favoured regions and over 8% residues were localized in additional allowed regions. Presence of residue over 90% in most favoured regions is the recognition for a good model. PDBsum was used for producing this plot. (B). 3D/1D profile of refined CAASPR-HM49 subtilisin like protease model. Above 81% residues displayed a score equal to or greater than 0.2 due to which the good model quality is strongly perceptible. (C). Z-score plot portraying how well the refined model aligns with experimentally generated structures. This plot compares the algorithm-generated models with X-ray diffraction and NMR solved protein structures having amino acid length similar to modelled protein. If the modelled protein lies outside the shaded area, then the model is probably integrated with errors. From the location of our modelled protein on this plot, it is highly evident that it is free from probable errors. In other words, the overall quality score of CAASPR-HM49 subtilisin like protease model is well within (not outside) the range.

This model portrayed 90.6% residues in most favoured regions, 8.9% in additional allowed regions, 0.3% in generously allowed regions (Fig 5A) reflecting the good quality of the model. Moreover, VERIFY3D evaluation also favoured model quality positively as minimum 80% residues should score equal to or beyond 0.2 in the 3D/1D profile, however, in our case 81.45% of the residues scored over or equal to 0.2 again attesting the quality of model (Fig 5B). Further, on PROSA plot, the model was found to be in agreement with the similar amino acid length proteins having empirically solved three dimensional structures (Fig 5C).

#### Lactose binds with high affinity to CAASPR-HM49 subtilisin like protease

Extra-exactitude flexible molecular docking score specifies favourable lactose-CAASPR-HM49 subtilisin like protease binding. Negative value of docking score indicates interactions between the two as a negative docking score of −7.108 was obtained in case of lactose in bound state with subtilisin like protease. The outcome of molecular docking was supplemented by results of MMGBSA. Negative value of binding free energy (−15.6014 kcal/mol) was estimated on subjecting the docked complex to MMGBSA energy calculations. Both flexible molecular docking and MMGBSA values infer feasible interaction between lactose and the active site residues of subtilisin like protease (Table 4).

**Table 4:** Final docking score and binding free energy values of lactose against CAASPR-HM49 subtilisin like protease.

Ligand name	Receptor	Docking score	Binding free energy value (kcal/mol)
Lactose	Subtilisin like serine protease	−7.108	−15.6014

#### Lactose-CAASPR-HM49 subtilisin like protease showed multiple interactions

**Fig 6:**
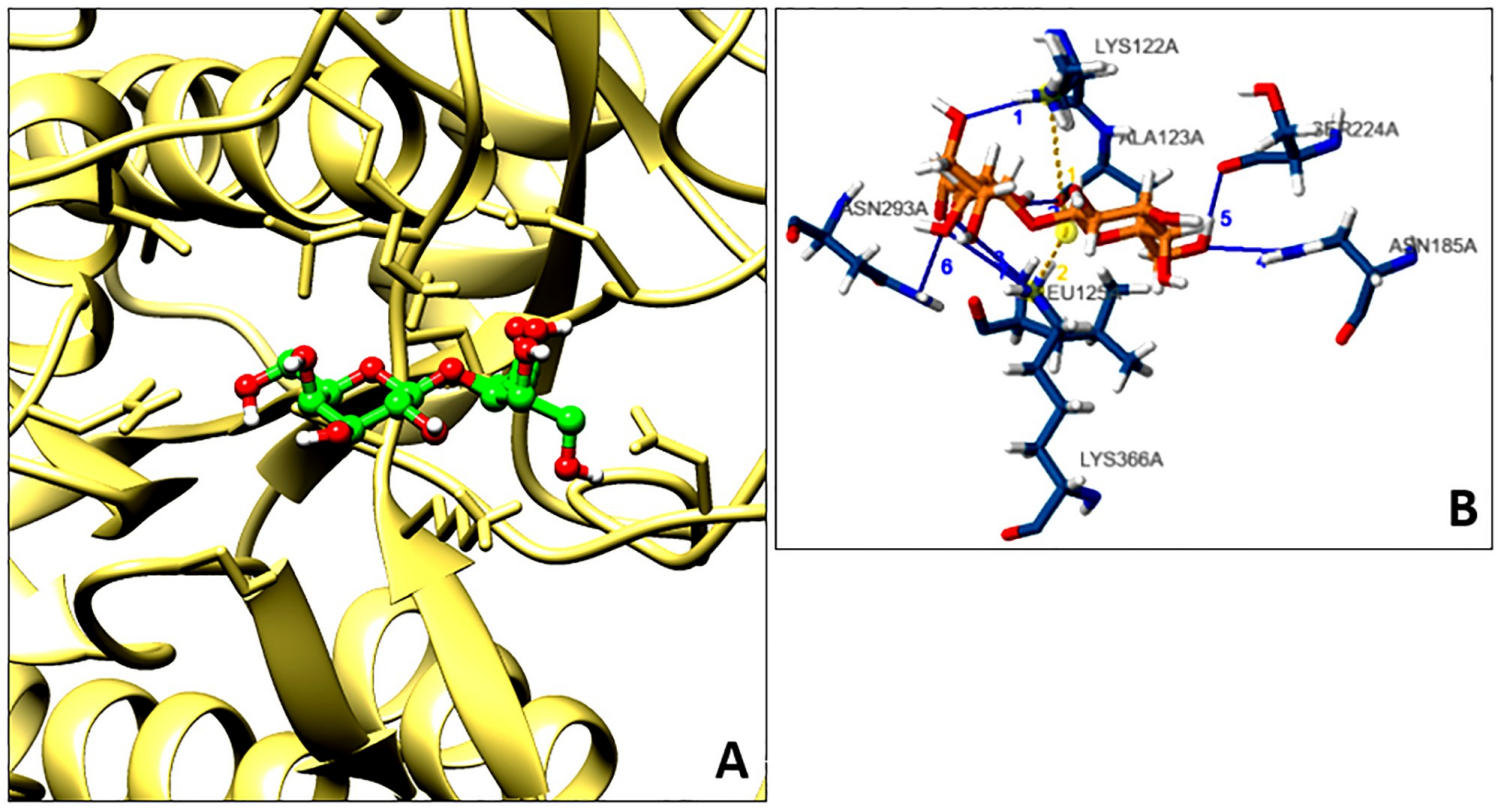
Three dimensional view of lactose in the binding pocket of CAASPR-HM49 subtilisin like protease (A). Interaction profile of lactose with various residues of this protease (B). Total seven hydrogen bonding interactions and two salt bridges exist between lactose and CAASPR-HM49 subtilisin like protease in docked state (blue solid lines represent hydrogen bonds and golden dotted lines depict salt bridges).

Various hydrogen bonding interactions and two salt bridges were seen between lactose and residues of CAASPR-HM49 subtilisin like protease. Totally, seven hydrogen bonds were seen in between the lactose-subtilisin like protease docked complex. Lysine 122, alanine 123, leucine 125, asparagine 185, lysine 366, serine 224 and asparagine 293 were among the residues of subtilisin like protease that were found to interact with lactose by way of hydrogen bonds. Two salt bridges occurred between the lactose and two residues—lysine 122 and lysine 366 of above-mentioned protease. This all specifies the stable interaction between the two and again certifies the findings of molecular docking and MMGBSA (Fig 6A and 6B).

### Application of CAASPR-HM49

#### Detergent compatibility and Blood stain removal test

**Fig 7:**
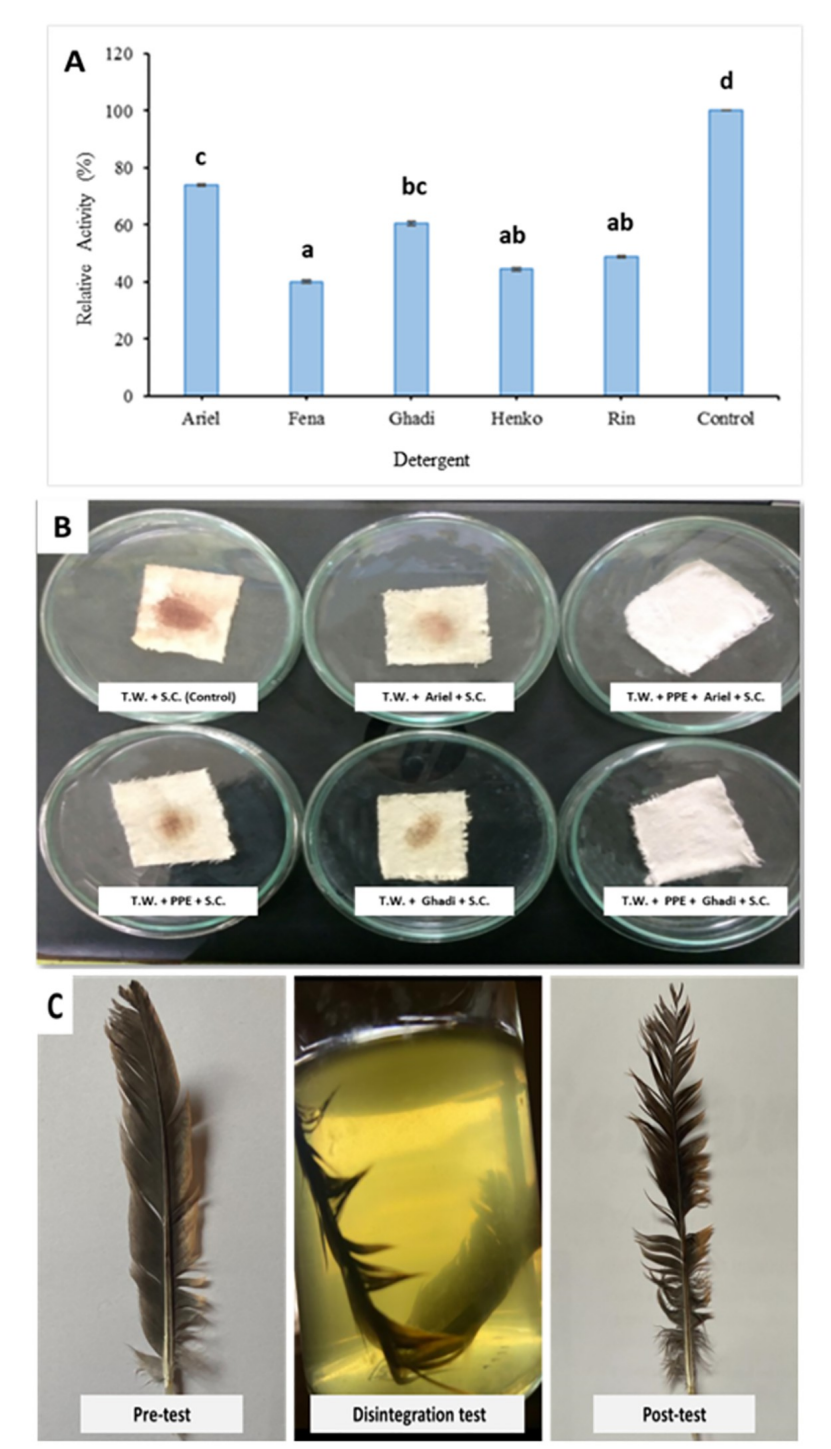
Commercial detergent compatibility test of protease from isolate HM49 (A). Different letters above the bars indicate significant enzyme activity at 0.05 significance level. Blood stain removal test of protease from isolate HM49 (B). Disintegration of chicken feather (C).

The results of compatibility test with some commonly used commercial detergents depicted that this protease was least compatible with Fena detergent in comparison to other tested detergents, retaining >73% of its activity significantly (p<0.05) with Ariel (Fig 7A) and thus could possibly be used as a detergent addictive in commercial detergent formulation. The effectiveness of this enzyme with Ariel was also confirmed by its washing performance removing blood stains completely which was also examined as a part of this study.

The stain removal study carried out at 20 °C for 20 min revealed that this protease enzyme efficiently removed recalcitrant blood stains when the treatment was augmented with enzyme in combination with detergents as compared to treatments having only tap water, enzyme or detergent solution which clearly indicate the assistance of this protease enzyme to the detergent resulting in its efficient cleaning action thus upgrading its washing performance (Fig 7B). However, among the tested detergents this enzyme was found to be more compatible with Ariel than Ghadi detergent as depicted by its complete stain removal.

#### Management of wastes: Chicken feather disintegration test

Disintegration test revealed that this cold-active protease was successful in hydrolysing the chicken feather to a great extent and could be utilized for prospective management of such wastes (Fig 7C).

## Discussion

Proteases are enzymes capable of catalysing the hydrolysis of the amide bonds linking together the amino acids into free amino acids or short peptides through a process called as ‘proteolysis’. They can be obtained from different organisms found almost in every habitat, with bacteria being one of them and these enzymes perform several functions in biology [49]. Microbes mainly *Bacillus* species are the main source of alkaline protease production, as these proteases possess such characteristics that make them fit for various industrial applications [12]. During this study, preliminary screening of collected soil samples for protease production depicted that more than half of the isolated bacterial isolates were protease positive supporting the influence of environment on the behaviour and status of the living organisms [50]. Biochemical characterization of *Bacillus velezensis* HM49 by carbohydrate utilization tests suggested its ability of consuming carbohydrates such as dextrose, fructose and trehalose which could possibly bring down the costs of fermentation as approximately 40% production costs of the industrially used enzymes is by far attributed to the use of growth medium, thus making it imperative to identify cost-effective media components that could be utilized for yielding the cold-adapted proteases [24]. A study conducted by Niyonzima, and More [51] supported this argument as they also concluded that the usage of cheap substrates like corn steep liquor and molasses—alternative sources for fructose, and trehalose as well as dextrose, and fructose respectively may lessen the production costs depending upon the media components by 30–40%. Antibiotic susceptibility test of this isolate concluded that it was sensitive to the antibiotics examined such as cephalosporin’s, penicillin’s, vancomycin and teicoplanin which inhibit the formation of cell-wall; aminoglycoside and macrolide antibiotics which inhibit the elongation of peptide chains at the time of protein synthesis. The isolate, HM49 also showed sensitivity towards fluoroquinolones inhibiting the nucleic acid synthesis. The present study which is the first report on this *Bacillus* sp. HM49 from the grassland soils of DNP situated in Zabarwan range of Western Himalaya thus leads to the isolation, multi-level identification and characterization of a psychro-trophic bacterial isolate, HM49 producing cold-active alkaline protease and possessing 99% sequence identity with *Bacillus velezensis*.

Upon successfully isolating and identifying the bacteria, HM49 with the highest proteolytic activity on the media plates employing different proteinaceous sources at various temperatures wherein its hydrolytic zone of clearance was checked every 24 h upto 72 h of incubation, with its pH, temperature, and incubation time being optimized using one variable at one-time approach so as to determine its optimal conditions for the proteolytic enzyme production. pH which mainly influences the redox potential and charged state of the microbial cells, thereby affects the microbial nutrient absorption and their enzyme secretion. pH optimization study revealed that the production increased from 6.0 reaching the maximum at 8.0 with a decline thereafter, clearly reflecting that the alkaline medium was required for the enzyme production [52]. However, various other studies reported the optimal pH for the production of protease at 7.0 [53], 9.0 [54], 10.0 [55], 11.0 [56]. *Bacillus* sp. HM49 was observed to yield maximum protease enzyme by incubating at 20 °C which was in accordance with a study on *Bacillus pumilus* isolated from glacial soils of Thajwas Glacier [57]. However, Nörnberg et al. [58] reported protease production from psychrotrophic isolates at 30 °C. Salwan et al. [59] while studying the prevalence of protease producing psychrotrophic bacterial diversity in Lahaul and Spiti provinces of the Western Himalayas incubated organisms at 28 °C for optimum protease generation. Likewise, the protease enzyme production by HM49 was observed to enhance with the increase in temperature reaching its maximal at 20 °C, however, any further elevation in the temperature caused a striking production decline, as the incubation temperature may influence its activeness as well as the stability profoundly. At its optimum temperature and pH, protease enzyme production reached highest after 72 h of incubation, which was in compliance with several studies [58, 59]. Although after 72 h, the enzyme production decreased drastically with any further extension in incubation time.

The partially purified enzyme (PPE) of *Bacillus* sp. HM49 procured after ammonium sulphate precipitation and dialysis was analysed to assess the effects of pH on its activity by varying pH (6, 7, 8, 9, 10, 11 and 12) in order to determine its optimum. The results affirmed that enzyme was active in between the pH of 6–12, with a reduction in enzyme activity towards the acidic side (i.e., pH 6) while as the highest activity was observed towards the alkaline side having an optimum at pH 8 thereby suggesting that the enzyme from isolate HM49 was an alkaline protease. This is in accordance with the conclusive work by Mushtaq et al. [37] on the temperature tolerant subtilisin-like serine alkaline protease from novel *Bacillus* sp. HM48. The temperature effects on enzyme activity of isolate HM49 was evaluated by placing the reaction mixture at various temperatures with every 5 °C interval ranging 5, 10, 15, 20, 25, 30, 35 and 40 °C. The results of which suggested the lowest activity was recorded at 5 °C, reaching its optimal activity at 20 °C beyond which the activity showed a declining trend. This optimal temperature of HM49 is much lower than the observed 40 °C by Yang et al. [60] while characterizing an alkaline protease from *Bacillus velezensis* SW5.

Stereochemical quality assessment of CAASPR-HM49 strongly favoured the model quality as above 90% residues are in most favoured region which indicates good model quality. Besides, VERIFY3D evaluation also favoured the model quality positively [37] as 81.45% of the residues scored over or equal to 0.2 against the standard minimum of 80% residues scoring equal to or beyond 0.2 in the 3D/1D profile. The quality of this model was further validated by PROSA plot that was found to be in accordance with the proteins having similar amino acid length [43]. Therefore, taken together, the distinct validation methods authenticated the quality of refined model for further studies. As far as the molecular docking study is concerned, negative docking score (−7.108) and negative value of binding free energy (−15.6014 kcal/mol) via of MMGBSA clearly revealed suitable interactions between the substrate, lactose and the active site residues of CAASPR-HM49 subtilisin like protease as it is believed that more negative the value, highly favourable is the interaction of ligand-receptor [46, 47].

As depicted from the figure (Fig 7) on detergent compatibility test, the CAASPR-HM49 was found to be stable with all of the tested commercial detergents. After 1h incubation, the highest activity of >73% was retained with Ariel, and the lowest (40%) with Fena thus suggesting its unique property that might be exploited in detergent industry, particularly for frail textiles such as woollens, silk etc. which are more prone to damage if washed at elevated temperature [61]. Although a study on protease from *B*. *safensis* strain S406 [62] revealed its stable nature in presence of few commercial detergents, but its optimum activity temperature value was 60 °C, which is much higher than isolate, HM49’s 20 °C, thus requiring high energy expenditure for water heating which ultimately increases the costs. The results of feather disintegration test for CAASPR-HM49 suggested that this enzyme can be utilized for the management of feather waste from slaughter houses, the protein hydrolysate of which could be exploited for organic fertilizer and fodder production [37].

## Conclusion

In this work, we successfully isolated cold-active protease producing psychrotrophic bacterial isolate, HM49 from the soils of a grassland site in DNP which was later identified as *Bacillus velezensis* HM49 whose preferred protease enzyme production time was about 72 h, at pH and temperature of 8.0 and 20 °C respectively that also happens to be the respective optimum pH and temperature (lowest reported for this *Bacillus* sp.) at which this enzyme depicted its maximal protease activity. An attempt was made to amplify CAASPR gene of HM49 in order to assess its type, molecular weight validation as well as functional applications via substrate docking studies. The enzyme when tested for its application depicted stability with several commercial detergents and also removed the blood stains in presence of detergent, Ariel thereby suggesting its potential as a detergent addictive. This enzyme also proved effective in disintegration of chicken feather. Thus, with such novel properties, the enzyme from isolate HM49 being active over a spectrum of temperatures (5 °C to 40 °C) and pH (6.0 to 12.0) suggests its potential mainly in green detergent industries focusing on environmentally sustainable laundry and cleaning demanding low to moderate heat and alkaline conditions as well as its utility in feather waste management.

## Supporting information

S1 Fig Morphological and biochemical characterization of *Bacillus* sp. HM49-pure culture on nutrient agar (A); Gram staining image showing Gram-positive rods (B); carbohydrate utilization tests (C); amylase test on starch agar (D); lipase test on tributyrin agar (E); Congo red test for cellulase (F).
(DOCX)

S2 Fig Original electrophoretic gel image of PCR amplicon of isolate, HM49 (marked in red) for 16S rRNA gene-based molecular identification. (DOCX)

S3 Fig Antibiotic susceptibility test of isolate, HM49. (DOCX)

S4 Fig Representative electrophoretic image of amplified CAASPR-HM49 gene based on its protein sequence. (DOCX)

S1 Table Antibiotic susceptibility test of isolate, HM49. (DOCX)

S2 Table Phenotypic identification of isolate, HM49. (DOCX)

S3 Table Carbohydrate utilization test of isolate, HM49. (DOCX)
