# Supplementary material for: Molecular and functional characterization of protease from psychrotrophic Bacillus sp. HM49 in North-western Himalaya
Source: PLoS One. 2023 Mar 30;18(3):e0283677. doi: 10.1371/journal.pone.0283677 (PMC10062638; doi:10.1371/journal.pone.0283677)
Supplement: S1 Fig — Morphological and biochemical characterization of Bacillus sp. HM49-pure culture on nutrient agar (A); Gram staining image showing Gram-positive rods (B); carbohydrate utilization tests (C); amylase test on starch agar (D); lipase test on tributyrin agar (E); Congo red test for cellulase (F). (DOCX) [file pone.0283677.s001.docx]

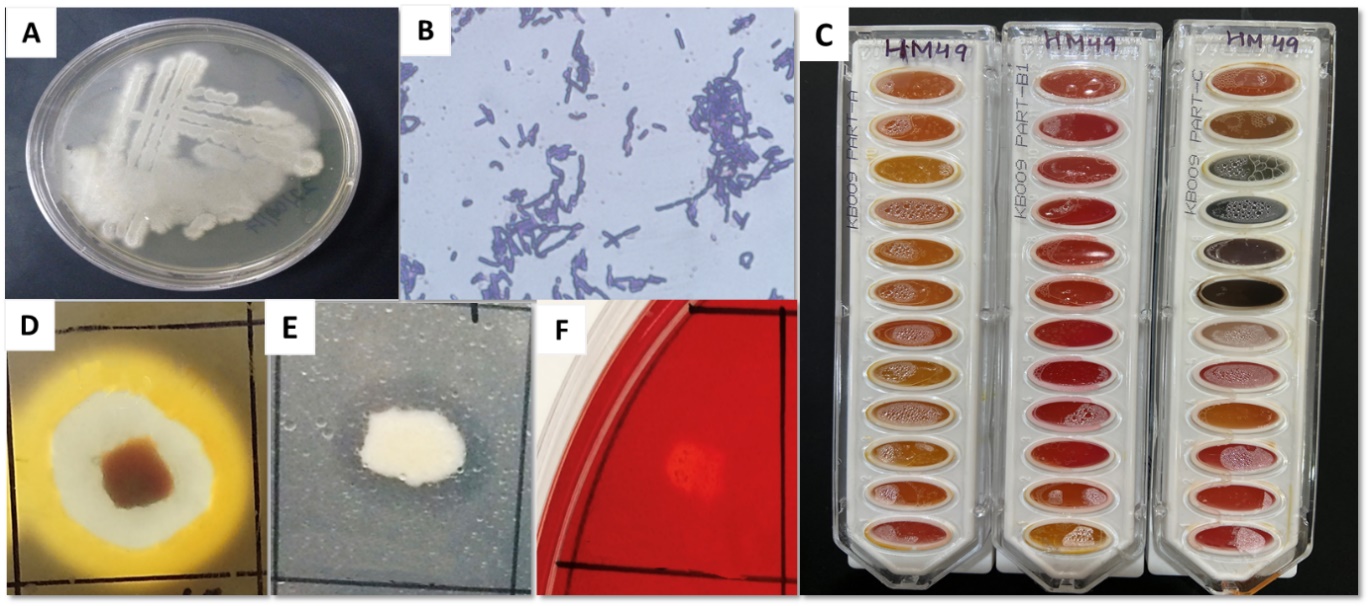


**S1 Fig. Morphological and biochemical characterization of *Bacillus* sp. HM49-pure culture on nutrient agar (A); Gram staining image showing Gram-positive rods (B); carbohydrate utilization tests (C); amylase test on starch agar (D); lipase test on tributyrin agar (E); Congo red test for cellulase (F).**
