# Supplementary material for: Molecular and functional characterization of protease from psychrotrophic Bacillus sp. HM49 in North-western Himalaya
Source: PLoS One. 2023 Mar 30;18(3):e0283677. doi: 10.1371/journal.pone.0283677 (PMC10062638; doi:10.1371/journal.pone.0283677)
Supplement: S2 Fig — (DOCX) [file pone.0283677.s002.docx]

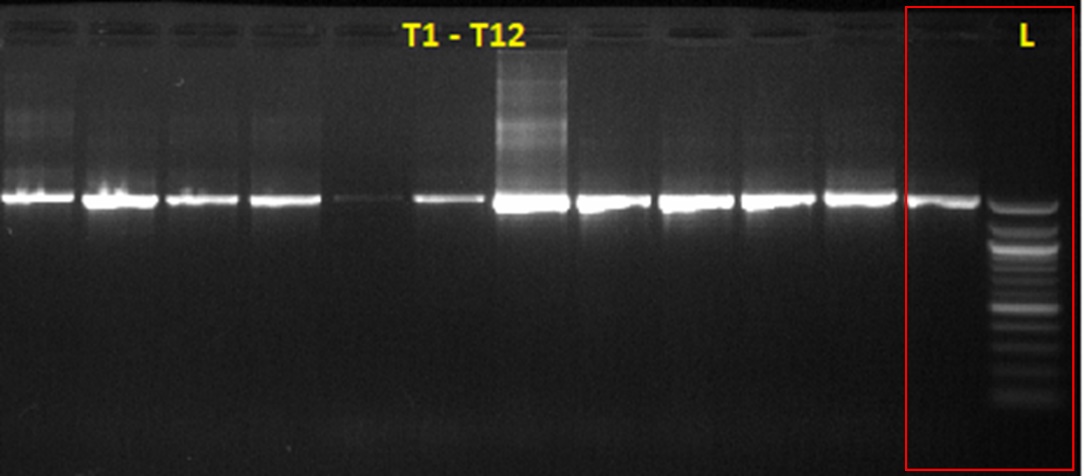


**S2 Fig. Original electrophoretic gel image of PCR amplicon of isolate, HM49 (marked in red) for 16S rRNA gene-based molecular identification.**
