# Supplementary figures and images for: Molecular and functional characterization of protease from psychrotrophic Bacillus sp. HM49 in North-western Himalaya
Source: PLoS One. 2023 Mar 30;18(3):e0283677. doi: 10.1371/journal.pone.0283677 (PMC10062638; doi:10.1371/journal.pone.0283677)

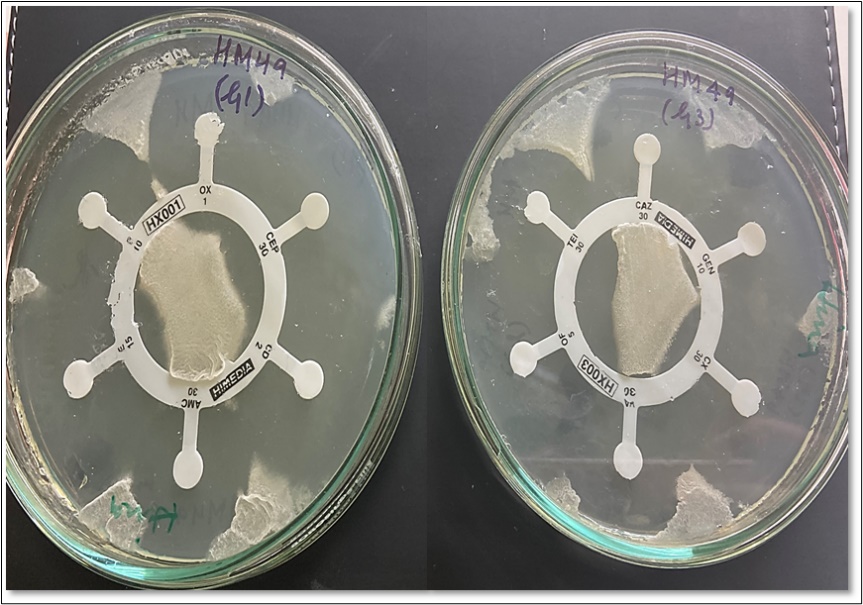


**S3 Fig. Antibiotic susceptibility test of isolate, HM49**

Supplement: S3 Fig — (DOCX) [file pone.0283677.s003.docx]
