# Supplementary material for: Molecular and functional characterization of protease from psychrotrophic Bacillus sp. HM49 in North-western Himalaya
Source: PLoS One. 2023 Mar 30;18(3):e0283677. doi: 10.1371/journal.pone.0283677 (PMC10062638; doi:10.1371/journal.pone.0283677)
Supplement: S4 Fig — (DOCX) [file pone.0283677.s004.docx]

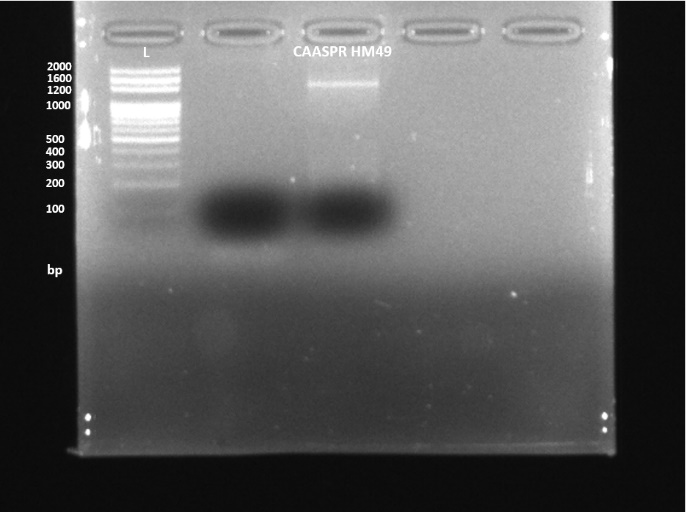


**S4 Fig. Representative electrophoretic image of amplified CAASPR-HM49 gene based on its protein sequence.**
