# Supplementary material for: Molecular and functional characterization of protease from psychrotrophic Bacillus sp. HM49 in North-western Himalaya
Source: PLoS One. 2023 Mar 30;18(3):e0283677. doi: 10.1371/journal.pone.0283677 (PMC10062638; doi:10.1371/journal.pone.0283677)
Supplement: S1 Table — (DOCX) [file pone.0283677.s005.docx]

**S1 Table. Antibiotic susceptibility test of isolate, HM49.**

| **Antibiotic** | **Concentration^*^** |
| --- | --- |
| Amoxiclav (AMC) | 30 mcg |
| Cefoxitin (CX) | 30 mcg |
| Ceftazidime (CAZ) | 30 mcg |
| Cephalothin (CEP) | 30 mcg |
| Clindamycin (CD) | 2 mcg |
| Erythromycin (E) | 15 mcg |
| Gentamicin (GEN) | 10 mcg |
| Ofloxacin (OF) | 5 mcg |
| Oxacillin (OX) | 1 mcg |
| Penicillin-G (P) | 10 units |
| Teicoplanin (TEI) | 30 mcg |
| Vancomycin (VA) | 30 mcg |

^*^Concentration of antibiotics according to Clinical and Laboratory Standards, CLSI (<https://clsi.org/>)
