# Supplementary material for: Molecular and functional characterization of protease from psychrotrophic Bacillus sp. HM49 in North-western Himalaya
Source: PLoS One. 2023 Mar 30;18(3):e0283677. doi: 10.1371/journal.pone.0283677 (PMC10062638; doi:10.1371/journal.pone.0283677)
Supplement: S2 Table — (DOCX) [file pone.0283677.s006.docx]

**S2 Table. Phenotypic identification of isolate, HM49.**

| **Macro-morphological colony characteristics** | |
| --- | --- |
| Appearance | Dull |
| Colour | Cream |
| Elevation | Flat |
| Margin | Entire |
| Size | Moderate |
| Shape | Circular |
| Texture | Rough |
| Transparency | Opaque |
| **Gram’s staining characteristics** | |
| Gram’s Reaction | Positive |
| Cell Shape | Bacilli |
