# Supplementary material for: Molecular and functional characterization of protease from psychrotrophic Bacillus sp. HM49 in North-western Himalaya
Source: PLoS One. 2023 Mar 30;18(3):e0283677. doi: 10.1371/journal.pone.0283677 (PMC10062638; doi:10.1371/journal.pone.0283677)
Supplement: S3 Table — (DOCX) [file pone.0283677.s007.docx]

**S3 Table. Carbohydrate utilization test of isolate, HM49.**

| **Positive Test Results** | **Negative Test Results** |
| --- | --- |
| Arabitol utilization | Adonitol utilization |
| Citrate utilization | Cellobiose utilization |
| Dextrose utilization | D-Arabinose utilization |
| Esculin hydrolysis | Dulcitol utilization |
| Fructose utilization | Erythritol utilization |
| Galactose utilization | Glycerol utilization |
| Inulin utilization | Inositol utilization |
| Lactose utilization | Malonate utilization |
| L-Arabinose utilization | Melizitose utilization |
| Maltose utilization | O-nitrophenyl ß-galactoside (ONPG) |
| Mannitol utilization | Rhamnose utilization |
| Mannose utilization | Salicin utilization |
| Melibiose utilization | Xylitol utilization |
| Raffinose utilization |  |
| Sodium gluconate utilization |  |
| Sorbitol utilization |  |
| Sorbose utilization |  |
| Sucrose utilization |  |
| Trehalose utilization |  |
| Xylose utilization |  |
| ɑ-Methyl-D-glucoside utilization |  |
| ɑ-Methyl-D-mannoside utilization |  |
